# Short-Term Intraocular Pressure Elevations after Combined Phacoemulsification and Implantation of Two Trabecular Micro-Bypass Stents: Prednisolone versus Loteprednol

**DOI:** 10.1155/2015/341450

**Published:** 2015-07-21

**Authors:** Qianqian Wang, Paul Harasymowycz

**Affiliations:** ^1^Department of Ophthalmology, Maisonneuve-Rosemont Hospital, Montreal, QC, Canada H1T 3W5; ^2^Montreal Glaucoma Institute, Montreal, QC, Canada H1V 1G5

## Abstract

*Objective*. To compare the effects of prednisolone and of loteprednol after combined phacoemulsification and trabecular micro-bypass stent implantation (phaco-iStent). *Methods*. Patients who underwent phaco-iStent between April 2013 and November 2014 were identified by retrospective chart review. Postoperatively, they received either prednisolone (*n* = 38) or loteprednol (*n* = 58). Baseline data was compared. Primary outcomes including intraocular pressure (IOP) and number of glaucoma medications (NGM) were analyzed at preoperative visit, postoperative day 1, weeks 1-2, weeks 3-4, and months 2-3. *Results*. Both groups had similar preoperative parameters (*p* > 0.05). The mean IOP spike occurred at postoperative weeks 1-2 with an increase of 2.21 ± 7.30 mmHg in the loteprednol group and 2.54 ± 9.28 mmHg in the prednisolone group. It decreased by weeks 3-4 in both groups and continued to improve at months 2-3. NGM showed significant reduction (*p* < 0.0001) after the surgery and remained stable in both groups. No significant group effect or time-group interaction in IOP and NGM evolution was detected (*p* > 0.05). The proportions of patients needing paracentesis were similar between the two groups. *Conclusion*. Similar early IOP elevations after combined phaco-iStent occurred with both prednisolone and loteprednol. Facilitated glucocorticoid infusion, altered aqueous humor outflow, and local inflammation may be contributing factors.

## 1. Introduction

The trabecular micro-bypass stent (iStent, Glaukos Corporation, Laguna Hills, CA) is a novel glaucoma procedure, usually performed in combination with phacoemulsification, for the treatment of mild to moderate open-angle glaucoma (OAG). An L-shaped titanium device, iStent, is inserted nasally into Schlemm's canal using a temporal clear corneal incision. It facilitates the outflow of aqueous humor by bypassing the trabecular meshwork (TM) and by maintaining a patent pathway between the anterior chamber and Schlemm's canal [1].

Previous studies reported short-term postoperative intraocular pressure (IOP) rise in patients who had received combined phacoemulsification and trabecular micro-bypass stent (phaco-iStent) [2–4], a phenomenon that we also observed in our practice.

The eye's vulnerability to damage from inflammation warrants routine use of topical glucocorticoids (GCs) for their anti-inflammatory effect after intraocular interventions. However, these medications are also associated with several side effects, including delayed wound healing, lowered resistance to infections, cataract formation, and increased IOP [5]. A retrometabolically designed topical GC [6], loteprednol, has similar therapeutic effect but decreased IOP response compared to conventional topical GCs [7, 8].

In this present study, we aim to characterize IOP elevation after combined phaco-iStent and to compare loteprednol's effects to those of prednisolone on IOP and the number of glaucoma medications (NGM).

## 2. Materials and Methods

This study was approved by the ethic committee at Maisonneuve-Rosemont Hospital (Montreal, QC).

Patients who were operated on by the second author for combined phaco-iStent procedure between April 2013 and November 2014 were identified using the EyeVu electronic medical record (Tecksoft, Mascouche, QC). A retrospective chart review was done for all patients to collect demographic and clinical data. The IOP and NGM at five different time points (preoperative visit, postoperative day (POD) 1, weeks 1-2, weeks 3-4, and months 2-3) were recorded.

Two iStents, one left-eye stent (model GTS100L) and one right-eye stent (model GTS100R), were inserted nasally into each eye after phacoemulsification. When possible, the stents were placed in areas of increased trabecular meshwork pigmentation in order to target preferential flow pathways near collector channels [9]. All patients received 250 mg of acetazolamide IV at the end of the intervention, followed by 250 mg of the same medication by mouth the same evening. Postoperatively, the first cohort (consecutive patients between April 2013 and December 2014) received a four-week tapering regimen of prednisolone (Pred Forte, Allergan), and the second (consecutive patients between January 2014 and November 2014) received loteprednol (Lotemax, Bausch & Lomb). Both groups also received a four-week tapering regimen of topical diclofenac (Voltaren, Novartis) and one week of topical moxifloxacin (Vigamox, Alcon).

The preoperative glaucoma medications were managed according to the same protocol in all patients. No washout of hypotensive medications preoperatively was required. Pilocarpine, if applicable, was stopped postoperatively in all cases. In patients with early to moderate glaucoma damage as per the Canadian Ophthalmological Society guidelines [10], topical prostaglandin analog, if applicable, was stopped postoperatively while the rest was continued. In patients with advanced glaucoma damage [10], all medications were continued postoperatively.

Basic demographic and clinical characteristics were compared between the two groups using Student's *t*-test and chi-square test. In patients with bilateral interventions, only the first operated eyes were included for the analysis. The two groups' evolution of IOP and NGM at the five different time points was studied using the mixed model for repeated measures (MMRM). When a significant temporal effect was detected, the Tukey-Kramer post hoc test was used to compare the means of the five time points in pairs. The proportions of patients with IOP elevation of ≥5 mmHg and ≥10 mmHg at specific time points and at all times were also compared between the two groups by chi-square test or Fisher's exact test when *n* ≤ 5.

Statistical analyses were done by SPSS (version 20, IBM, New York) and by SAS (version 9.3, SAS institute, North Carolina), using 5% as the threshold level of significance.

## 3. Results

We identified 96 patients, 38 in the prednisolone group and 58 in the loteprednol group. Preoperative demographic and clinical parameters were similar between the two groups (see Table 1).

Transitory IOP elevations mainly occurred around weeks 1-2, with a mean IOP increase of 2.21 ± 7.27 (standard deviation) mmHg in loteprednol and 5.54 ± 9.28 mmHg in prednisolone. At subsequent visit of weeks 3-4, IOP improved with a mean reduction of 2.79 ± 6.08 mmHg in loteprednol and of 1.57 ± 6.95 mmHg in prednisolone, maintained at 3.49 ± 5.23 mmHg and 1.93 ± 6.58 mmHg, respectively, at final visit of months 2-3.

**Figure 1:**
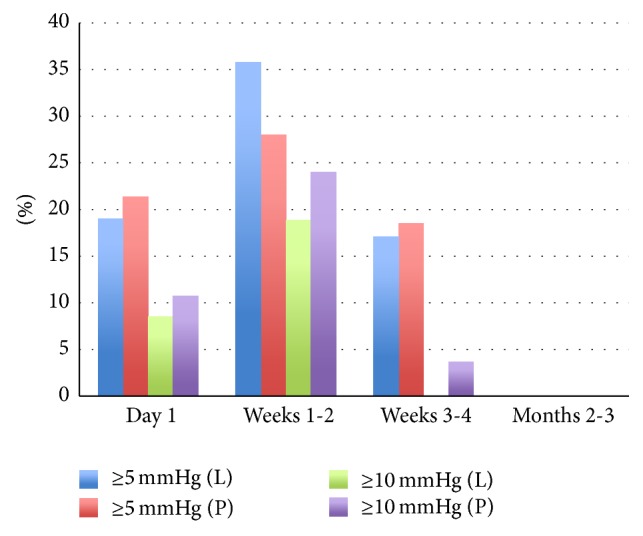
Percentages of eyes with categorical IOP elevations of ≥5 mmHg and of ≥10 mmHg.

**Figure 2:**
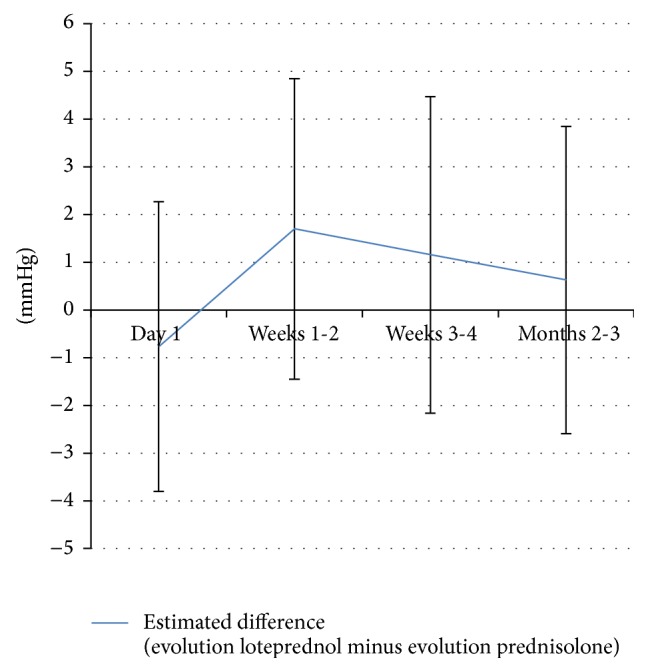
Estimated IOP evolution difference (evolution loteprednol minus evolution prednisolone), adjusted for preoperative IOP difference. For instance, the IOP evolution of loteprednol was 0.7676 mmHg lower than that of prednisolone on day 1.

Postoperatively, the number of paracenteses performed to temporarily decrease IOP was similar in both groups (*p* = 0.374). The proportions of patients with categorical IOP elevation of ≥5 mmHg and of ≥10 mmHg over baseline (see Figure 1) did not differ significantly between the two groups at any point of time (*p* > 0.05). MMRM analysis did not show significant interaction between group and time (*p* = 0.7980), or a significant effect of group (*p* = 0.1134). There was however a significant time effect (*p* < 0.0001) for both groups, with important IOP elevations at weeks 1-2 comparing to other time points (*p* < 0.001). Estimation of IOP evolution (time-specific IOP values minus preoperative values) difference at each time point between the two groups, adjusted for preoperative IOP difference, is shown in Figure 2.

The preoperative mean NGM of 2.23 ± 1.44 (loteprednol) and 2.00 ± 1.31 (prednisolone) showed a reduction of 1.56 ± 0.68 and 1.36 ± 0.77, respectively, on postoperative day 1. The mean NGM reduction was maintained at 1.31 ± 0.56 for loteprednol and 1.5 ± 1.23 for prednisolone at months 2-3. No significant effect of group (*p* = 0.0787) or interaction between group and time (*p* = 0.3253) was detected by MMRM analysis. A significant time effect (*p* < 0.0001) was demonstrated by the Tukey-Kramer post hoc test, with NGM at all postoperative time points being significantly lower than the preoperative values (*p* < 0.0001).

## 4. Discussion

Combined phaco-iStent procedure was previously shown to provide a mild to moderate IOP reduction and medication sparing effects. In addition to the well-recognized mid-to-long-term IOP-lowering effect from phacoemulsification alone [11], recent analysis on iStent as a solo procedure also confirmed a sustained, statistically significant hypotensive effect [12]. Our results were similar to previous reported numbers (see Table 2). However, the follow-up period in this current study was shorter given its goal of studying short-term postoperative IOP elevation, limiting the comparison.

In our study, despite a trend suggesting lower preoperative IOP in the prednisolone group, the values were not significantly different (*p* = 0.3715) between the two groups. The MMRM analysis demonstrated no significant interaction between group and time or significant group effect for IOP and NGM, suggesting the impact of loteprednol was similar to that of prednisolone over the studied period of 3 months. In fact, except for POD 1, IOP readings adjusted for the preoperative difference tended to be slightly more elevated in the loteprednol group, even though such elevation was not significant (see Figure 2).

Short-term postoperative IOP elevations in patients undergoing phaco-iStent have been previously reported by other studies. The presence of residual viscoelastic as well as stent malposition and obstruction were reported as causes of short-term IOP rise [2, 4, 13]. Fea also mentioned “a few cases of a slight postoperative IOP increase” without further details but suggested that such increase had been “reported after cataract surgery” [3]. In our study, stent obstruction by iris was occasionally seen in patients but did not lead to IOP elevation. No subsequent intervention was therefore required. Another important factor to be considered in postoperative IOP elevation is the management of glaucoma medications. As topical medications remain to be the first-line treatment of OAG, many patients require at least one class of medication, if not more, to achieve target IOP control preoperatively. Depending on the medication class, one would expect a 20–35% reduction in IOP though the additive IOP-lowering efficacy is less when medications are used in combination [14, 15]. Even though glaucoma surgeries offer additional IOP lowering and may allow topical medication reduction, it is possible to have short-term postoperative IOP rebound if the patient's preoperative glaucoma medications are reduced too aggressively. In our study, we systematically stopped pilocarpine and/or prostaglandin analogs depending on the patient's glaucoma damage (see Section 2). As first-line medical treatment for OAG, prostaglandin analogs increase outflow through the IOP-independent uveoscleral pathway, representing between 12 and 54% of the total aqueous outflow [14]. On the other hand, pilocarpine enhances the traditional trabecular outflow through its direct cholinergic parasympathomimetic action but is infrequently used nowadays for OAG treatment. In our patients, the cessation of these agents could theoretically result in a diminution of aqueous outflow but should be counterbalanced by the direct aqueous access into the Schlemm's canal through the two iStents, bypassing the trabecular meshwork.

An association between the use of topical GCs and IOP elevation has been recognized for over 50 years. Steroid-induced IOP elevation tends to occur after 2–4 weeks of topical GCs use [5, 16]. Suggested mechanisms include impaired TM cell function, increased extracellular matrix (ECM) deposition and cytoskeleton size of TM, and altered gene expression, such as the induction of myocilin [16]. Several studies have noted decreased IOP response with loteprednol [7, 8], possibly due to its rapid metabolism which deactivates the medication before it can reach GC receptors [5]. However, we found similar IOP and NGM evolution in both groups (see above) and that IOP elevation was not less frequent with loteprednol. It is possible that the surgically created direct opening between the anterior chamber and Schlemm's canal may have facilitated GC infusion and allowed the unmetabolized loteprednol to bind to GC receptors, which in turn lead to subsequent structural and biochemical changes in the TM and secondary IOP elevation. We also suspect that altered aqueous humor outflow dynamics as well as local inflammation following surgical manipulation in the TM may be contributing factors of this transient postoperative IOP rise in glaucomatous eyes. Future histology studies are needed to confirm such changes.

The study is limited by its retrospective nature and the lack of randomization. However, both groups consisted of consecutive patients over sequential time periods in order to decrease selection bias. There is a trend suggesting higher baseline IOP in the loteprednol group, which may represent more recalcitrant disease. However, no statistically significant difference was found among preoperative characteristics between the two groups. Moreover, the MMRM analysis on IOP and NGM did not show significant group-time interaction, suggesting that there is no significant difference in terms of the evolution of these outcomes between the two groups.

## 5. Conclusion

Combined phacoemulsification and trabecular micro-bypass stent provide mild to moderate IOP reduction and NGM sparing effect. Postoperatively, patients may develop transitory IOP elevations, especially around weeks 1-2, and patients with more advanced glaucomatous disease should be carefully monitored. Our study has found similar evolution with postoperative prednisolone and with loteprednol. One hypothesis is that the surgically created pathway between the anterior chamber and Schlemm's canal may have facilitated infusion and receptor binding of unmetabolized loteprednol, which lead to secondary IOP elevation. Another hypothesis is that the IOP elevations are unrelated to steroid use and that local inflammation as well as altered aqueous humor outflow dynamics may explain the phenomenon. Further studies, including use of NSAID only after trabecular bypass stents surgery, may help in determining the etiology of these transitory IOP elevations.

## Figures and Tables

**Table 1:** Preoperative clinical and demographic characteristics.

Baseline characteristics	Prednisolone	Loteprednol	*p* value
Laterality			
OD	58%	57%	0.923
Sex			
Female	58%	53%	0.668
Race			
Asian	0%	2%	0.474
Caucasian	95%	86%	
Hispanic	0	3%	
Black	5%	9%	
Age (years)			
Mean	73 ± 8	69 ± 11	0.073
Visual field (dB)			
Mean deviation	−5.41 ± 5.66	−8.48 ± 6.50	0.054
Pachymetry (microns)	542 ± 41	544 ± 43	0.806
Pre-op IOP (mmHg)	15.62 ± 4.53	17.57 ± 5.25	0.3715
Pre-op NGM	2.00 ± 1.31	2.23 ± 1.44	0.440
Pre-op BCVA (logMAR)	0.26 ± 0.22	0.29 ± 0.33	0.664

BCVA: best corrected visual acuity.

**Table 2:** IOP and NGM reductions reported in previous studies and current study.

Study	Number	Topical steroid	Follow-up (mo)	Mean IOP reduction (mmHg) ± SD	Mean NGM reduction ± SD
Spiegel et al. [13]	58	NA	12	4.4 ± 4.54	1.2 ± 0.7

Fea [3]	12	NA	15	3.2 ± 3.0	2.0 ± 0.9

Samuelson et al. [2]	106	Prednisolone	12	1.5 ± 3.0	1.4 ± 0.8

Arriola-Villalobos et al. [4]	19	Dexamethasone	53.68 ± 9.26	3.16 ± 3.9	0.47 ± 0.96

Current study	96	—	3	2.50 ± 5.80	1.38 ± 1.43
	38	Prednisolone		1.93 ± 6.58	1.5 ± 1.23
	58	Loteprednol		3.49 ± 5.23	1.31 ± 0.56

SD: standard deviation.

## References

[1] Francis B. A., Singh K., Lin S. C. (2011). Novel glaucoma procedures: a report by the American Academy of Ophthalmology. *Ophthalmology*.

[2] Samuelson T. W., Katz L. J., Wells J. M., Duh Y.-J., Giamporcaro J. E. (2011). Randomized evaluation of the trabecular micro-bypass stent with phacoemulsification in patients with glaucoma and cataract. *Ophthalmology*.

[3] Fea A. M. (2010). Phacoemulsification versus phacoemulsification with micro-bypass stent implantation in primary open-angle glaucoma: randomized double-masked clinical trial. *Journal of Cataract and Refractive Surgery*.

[4] Arriola-Villalobos P., Martínez-de-la-Casa J. M., Díaz-Valle D., Fernández-Pérez C., García-ánchez J., García-Feijoó J. (2012). Combined iStent trabecular micro-bypass stent implantation and phacoemulsification for coexistent open-angle glaucoma and cataract: a long-term study. *British Journal of Ophthalmology*.

[5] Comstock T. L., Decory H. H. (2012). Advances in corticosteroid therapy for ocular inflammation: loteprednol etabonate. *International Journal of Inflammation*.

[6] Bodor N., Loftsson T., Wu W. (1992). Metabolism, distribution, and transdermal permeation of a soft corticosteroid, loteprednol etabonate. *Pharmaceutical Research*.

[7] Novack G. D., Howes J., Crockett R. S., Sherwood M. B. (1998). Change in intraocular pressure during long-term use of loteprednol etabonate. *Journal of Glaucoma*.

[8] Holland E. J., Bartlett J. D., Paterno M. R., Usner D. W., Comstock T. L. (2008). Effects of loteprednol/tobramycin versus dexamethasone/tobramycin on intraocular pressure in healthy volunteers. *Cornea*.

[9] Hann C. R., Fautsch M. P. (2009). Preferential fluid flow in the human trabecular meshwork near collector channels. *Investigative Ophthalmology & Visual Science*.

[10] Rafuse P. E., Buys Y. M., Damji K. F. (2009). Canadian ophthalmological society evidence-based clinical practice guidelines for the management of glaucoma in the adult eye. *Canadian Journal of Ophthalmology*.

[11] Poley B. J., Lindstrom R. L., Samuelson T. W., Schulze R. (2009). Intraocular pressure reduction after phacoemulsification with intraocular lens implantation in glaucomatous and nonglaucomatous eyes. Evaluation of a causal relationship between the natural lens and open-angle glaucoma. *Journal of Cataract and Refractive Surgery*.

[12] Malvankar-Mehta M. S., Chen Y. N., Iordanous Y. (2015). iStent as a solo procedure for glaucoma patients: a systematic review and meta-analysis. *PLoS ONE*.

[13] Spiegel D., Wetzel W., Neuhann T. (2009). Coexistent primary open-angle glaucoma and cataract: interim analysis of a trabecular micro-bypass stent and concurrent cataract surgery. *European Journal of Ophthalmology*.

[14] Costagliola C., Dell'Omo R., Romano M. R., Rinaldi M., Zeppa L., Parmeggiani F. (2009). Pharmacotherapy of intraocular pressure—part II. Carbonic anhydrase inhibitors, prostaglandin analogues and prostamides. *Expert Opinion on Pharmacotherapy*.

[15] Costagliola C., Dell'Omo R., Romano M. R., Rinaldi M., Zeppa L., Parmeggiani F. (2009). Pharmacotherapy of intraocular pressure: Part I. Parasympathomimetic, sympathomimetic and sympatholytics. *Expert Opinion on Pharmacotherapy*.

[16] Clark A. F., Wordinger R. J. (2009). The role of steroids in outflow resistance. *Experimental Eye Research*.

